# Environmental DNA: The First Snapshot of the Vertebrate Biodiversity in Three Sicilian Lakes

**DOI:** 10.3390/ani13233687

**Published:** 2023-11-28

**Authors:** Manuela Mauro, Mario Lo Valvo, Mirella Vazzana, Slobodanka Radovic, Aiti Vizzini, Rosario Badalamenti, Lucie Branwen Hornsby, Vincenzo Arizza

**Affiliations:** 1 Dipartimento di Scienze e Tecnologie Biologiche, Chimiche e Farmaceutiche (STEBICEF), Università degli Studi di Palermo, Via Archirafi, 18, 90123 Palermo, Italy; mario.lovalvo@unipa.it (M.L.V.); aiti.vizzini@unipa.it (A.V.); rosario.badalamenti@unipa.it (R.B.); luciebranwen.hornsby@unipa.it (L.B.H.); vincenzo.arizza@unipa.it (V.A.); 2 IGA Technology Services Srl., Via Linussio, 51, 33100 Udine, Italy; sradovic@igatechnology.com

**Keywords:** eDNA, terrestrial vertebrates, freshwater vertebrates, freshwater environment, monitoring

## Abstract

**Simple Summary:**

Freshwater ecosystems are important for global diversity and are subject to anthropogenic impacts. Knowing the biodiversity of these sites is important, and a revolutionary method to survey this is currently the use of environmental DNA (eDNA) released by organisms into the environment. In this study, eDNA evaluation was used to analyze water samples obtained from Lake Poma, Piana degli Albanesi Lake and Lake Scanzano. The results showed that by using eDNA, it was possible to provide the first snapshot of vertebrate biodiversity in these three lakes. Moreover, the results also showed that eDNA could be a useful tool to evaluate the ecology of the environment.

**Abstract:**

Freshwater ecosystems play a key role in global diversity and are subject to a series of anthropic impacts, often leading to biodiversity loss. The organisms inhabiting these sites continuously release DNA into the environment through cells, excrement, gametes and/or decomposing matter; thus, evaluation of this eDNA could revolutionize the monitoring of biodiversity. In this study, environmental DNA metabarcoding was used for the first time in three Sicilian lakes: Lake Poma, Piana degli Albanesi Lake and Lake Scanzano. Results obtained provide the first snapshot of vertebrate biodiversity in these three lakes, where little is known, to provide valuable information useful for creating a baseline of knowledge regarding the biodiversity in these three lakes. Another important result was the detection of marine species, most likely due to some kind of anthropogenic contamination. Environmental DNA is a useful tool to evaluate both biodiversity and the ecological status of the environment; it has the potential to complement traditional methods, and the use of both approaches may offer a more comprehensive understanding of the ecosystem.

## 1. Introduction

Freshwater ecosystems are of fundamental importance as they play a key role in ensuring global diversity and provide invaluable goods and services for various forms of life on Earth [1,2,3,4,5,6,7]. These environments are currently undergoing substantial change due to anthropic impact. Although little studied, they are most affected by the creation of cultivated land, which inevitably leads to the fragmentation and/or destruction of natural habitats and water pollution [7,8,9,10,11,12,13,14]. In addition, rapid urbanization and industrialization processes (wastewater from agriculture or households) are dictating significant changes [15]. Lastly, it is important to note that changes in land use can alter the inputs of river systems and therefore affect aquatic communities by reducing the supply of terrestrial carbon and influencing the retention of organic matter, thereby leading to the decline of aquatic biodiversity [7,16,17,18,19]. For these reasons, it is extremely important to learn as much as possible about the species that live in freshwater environments, analyzing both single groups of organisms, such as fish, algae or invertebrates, and entire multitrophic communities [17,20,21]. In this context, biomonitoring is known to be fundamental for ecological assessment and is at the basis of environmental protection [22]. In recent years, the monitoring technique which uses environmental DNA has become increasingly widespread, revolutionizing traditional methods of biomonitoring, especially in freshwater ecosystems [7,23,24,25,26,27]. 

Some authors claim that traditional methods of identification based on morphology are often too costly in terms of time, work, disturbance of the habitat and even difficulty in finding the required taxonomic skills. For these authors, a metabarcoding technique applied to environmental samples, such as DNA released into the environment, would overcome these obstacles by providing large-scale spatial resolution and high-quality levels of biomonitoring [24,28]. 

This technique consists the extraction and analysis of genetic material released into the environment through feces, saliva, urine and skin cells, obtained directly from the environmental samples of water, soil, sediment or ice [29]. The method relies on the detection of a short fragment of DNA for the identification of the species and different taxa using the “DNA barcode”, a standardized DNA region [30,31]. It is a technique which has already been applied to the monitoring of aquatic communities (e.g., microbes, algae, macroinvertebrates, fish and mammals), demonstrating its ability to identify the presence of rare, invasive, extinct species or those difficult to detect using conventional methods [31,32,33,34,35,36,37,38,39,40,41,42]. With specific reference to the Region of Sicily, knowledge of the species present in freshwater environments is currently highly fragmentary (e.g., based on information from the population) and no official scientific documents linked to the sites describe vertebrate biodiversity exhaustively. In the light of human impact and climate change today, this information is fundamental in the provision of a baseline to understand if change will occur at a biodiversity level. This study focused on three Sicilian Lakes: Lake Poma, Lake Scanzano and Lake Piana degli Albanesi, three important artificial basins which supply fresh water to the urban centers of the area [43]. Due to their naturalistic importance, in 1994, Lake Poma and, in 1999, Lake Piana degli Albanesi were recognized by the Sicilian Region as Protection Oasis and Wildlife Refuge. For the same reason, Lake Piana degli Albanesi is a protected area under the Habitats Directive (ITA020013 Lake of Piana degli Albanesi). In particular, we applied the environmental DNA technique to detect the taxa and characterize the vertebrate coenoses in these lakes, where knowledge of vertebrate biodiversity is currently scarce or largely undocumented. In particular, the aims of this research within these three wetlands were to use eDNA to have the first snapshot of vertebrate biodiversity in these three lakes, where little is known to provide valuable information useful for creating a baseline of knowledge regarding the biodiversity in these three lakes.

## 2. Materials and Methods

### 2.1. Study Area

#### Lakes Description

**Figure 1:**
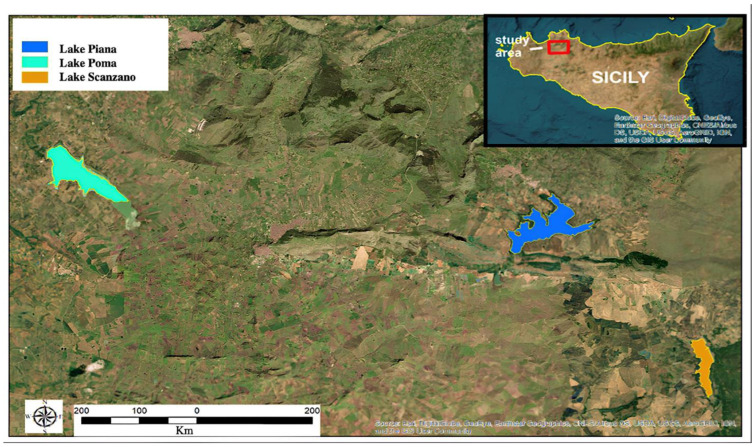
Sicilian lakes in which eDNA was evaluated.

Three artificial basins located in the province of Palermo were selected for sampling: Lake Poma, Lake Scanzano and Piana degli Albanesi Lake (Figure 1). All the lakes analyzed have dams. The first is located in Partinico (37°59′17.45″ N–13°6′6.76″ E) at an altitude of 198 m above sea level (asl). It covers an area of approximately 268 hectares and has a perimeter of 11.1 km; the second is located in Piana degli Albanesi (hereinafter referred to as called Lake Piana, 37°58′20.54″ N–13°17′58.34″ E) at an altitude of 606 m asl, has an area of approximately of 289 hectares and a perimeter of 16.6 km; the third is located in Piana degli Albanesi (and only partially in the municipality of Monreale, 37°55′31.84″–13°22′7.55″ EN) at an altitude of 518 m asl, with an area of 101 hectares and a perimeter of 7.6 km. All three lakes are primarily situated in agricultural areas, with the water being used for both irrigation and to supply a number of urban centers with fresh water. The lakes are fed partly by rainwater and partly by influx from rivers [43].

In Table 1, the different types of land use are listed (Corine Biotopes; carta HABITAT 1:10.000″ of the Regione Siciliana) together with their surface area in hectares by applying a buffer of 1 km with respect to the perimeter of each lake.

### 2.2. Water Sampling and eDNA Extraction

The water samples were collected in the months of October and November 2022 from Lake Poma (UTMWGS84 333224,4206239), Lake Piana (UTMWGS84 350642,4204113) and Lake Scanzano (UTMWGS84 3566384198788). Each lake was sampled in three different points that being influx to the lake, confluence in the dam (there is a dam in all lakes) and halfway. For each point mentioned, the sampling was done in the middle of the water column and repeated three times, sampling 2 L for each replicate using sterile glass bottles (autoclaved and cleaned also using a 10% HCl acid-rinse). During transportation, the water samples were kept in a cool and dark place. After reaching the laboratory of the STEBICEF Department at the University of Palermo, the samples were vacuum-filtered in a sterile environment (vacuum filter systems HC Series, Cheimika-HC/SLGS/F05002, Pellezzano, Italy), using nitrocellulose membranes (MF-Millipore, 0.22 µm MCE Membrane, 47 mm, Merck, GSWP04700, Darmstadt, Germany). More specifically, one filter was used for each 2 L replicate. As a control sample, MilliQ water was filtered using other filters to monitor contamination during the filtration process. Several decontamination precautions were used, including UV light. Vacuum filtration funnels, tweezers, scissors and the filter processing environment were also cleaned using 10% bleach and 96% ethanol. In accordance with Thomsen et al. [42], each filter was cut into small 1 mm strips to facilitate the eDNA extraction process, which was performed using DNeasy Blood & Tissue Kits (Qiagen). Each eDNA sample was stored at −20 °C.

### 2.3. eDNA Library Preparation and Bioinformatics Analysis

Metabarcoding analysis of eDNA samples was performed by IGA Technology Services s.r.l. (Udine, Italy) The PCR amplification consisted of two steps; the first step was performed with primers that amplified 106 bp from the 12S rRNA region [44]. 

The PCR mix in the final volume of 25 µL contained 12.5 µL 2 × KAPA HiFi HotStart ReadyMix (Roche, Wilmington, MA, USA), 2.5 µL of the forward primer 12SV5-F 5′-ACTGGGGATTAGATACCC-3′ (with Illumina adaptor 2 µM) and 2.5 µL of the reverse primer 12SV5-R 5′-TAGAACAGGCTCCTCTAG-3′ (with Illumina adaptor 2 µM). After adding 50 ng of the DNA extract, this mix was incubated using the following PCR conditions: initial denaturation for 3 min at 95 °C, 30 cycles of denaturation for 30 s at 95 °C, annealing for 30 s at 55 °C and extension for 30 s at 72 °C and a final extension for 4 min at 72 °C.

PCR products were purified using 1.6X Ampure XP beads (Beckman Coulter Life Sciences, Indianapolis, IN, USA) and eluted in 35 µL Tris-HCl pH 8.0 buffer. For the second step (the index PCR), 7.5 µL of the purified PCR product was added to a PCR mix containing 12.5 µL 2 × KAPA HiFi HotStart ReadyMix (Roche, Wilmington, MA, USA) and 2.5 µL of each index primer Nextera XT (Illumina, San Diego, CA, USA). PCR conditions for the index PCR were: initial denaturation for 3 min at 95 °C, 9 cycles of denaturation for 30 s at 95 °C, annealing for 30 s at 55 °C and extension for 30 s at 72 °C and a final extension for 5 min at 72 °C. After measuring with the Qubit 1X dsDNA HS Assay Kit (Thermo Fisher Scientific, Waltham, MA, USA), the indexed PCR products were equimolarly pooled and sent for sequencing using the Illumina MiSeq 2 × 300 bp platform (Illumina, San Diego, CA, USA). Base calling, demultiplexing and adapter masking were performed on instruments with the MiSeq Reporter.

An internal pipeline was created to analyze the metabarcoding sequences. Where the amplicon length was permissive with respect to the read sequencing length, 3′-ends of pairs were overlapped with flash v. 1.2.11 [45] and parameters “--max-overlap 70 --min-overlap 8” (to generate consensus pseudo-reads), while non-overlapping reads were maintained as separated pairs. We retained both overlapping and non-overlapping reads. Primer sequences used to amplify the variable 12S region were removed, with cutadapt v. 2.7 [46] and parameters: “--discard-untrimmed --minimum-length 70 --overlap 10 --times 2 --error-rate 0.15”. Reads were retained if they maintained a minimum length of 70 bp. Low-quality bases at 3′ tails of reads were trimmed with the erne-filter v. 1.4.3 [47] and parameters: “--min-size 70”. The QIIME pipeline v. 1.9.1 [48] was then executed. The library was scanned for the presence of chimeras with the VSEARCH algorithm v. 2.14.1 [49]. The Operational Taxonomic Unit (OTU) picking process was performed in “open-reference” mode against the 12S Vertebrate Reference Set for the RDP Classifier release v2.0.0 database https://zenodo.org/badge/latestdoi/391459819 [50,51,52]. Reference sequences were obtained from the NCBI nucleotide database (accessed on July 2021) and MitoFish (accessed on March 2020). This version contains 19,654 reference sequences and 15,007 taxa at all ranks, including 9564 species. Taxonomy is based on the NCBI taxonomy database. Taxonomy was assigned to OTUs using the pre-defined taxonomy mapping file of the reference sequences, with the RDP classifier v. 2.2 [51]. Only OTU matching with 97% minimum identity threshold and with minimum confidence threshold of 0.50 were retained and subjected to further classification.

### 2.4. Data Analysis

Using exclusively the qualitative–quantitative list of the wild species identified by the eDNA of this study, in the ecosystems of these three Sicilian lakes, the specific richness values (S) and the biodiversity indices (H′) calculated with the Shannon algorithm were determined. The coenoses found in the three lakes were then compared, by calculating the values of both qualitative similarities (with the Sorensen index) and qualitative–quantitative similarities (with the Bray–Curtis and Morisita index). To correct the significant asymmetry in the number of fragments found among different vertebrate taxa, we conducted a base-10 logarithmic transformation of the fragment counts for biodiversity and similarity calculations.

## 3. Results

**Figure 2:**
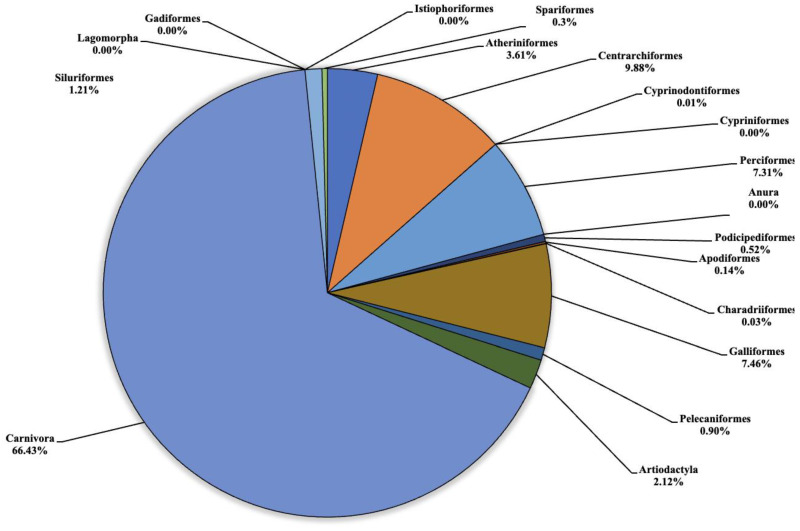
Pie chart (expressed as a percentage) representing the taxonomic orders identified in Lake Poma.

**Figure 3:**
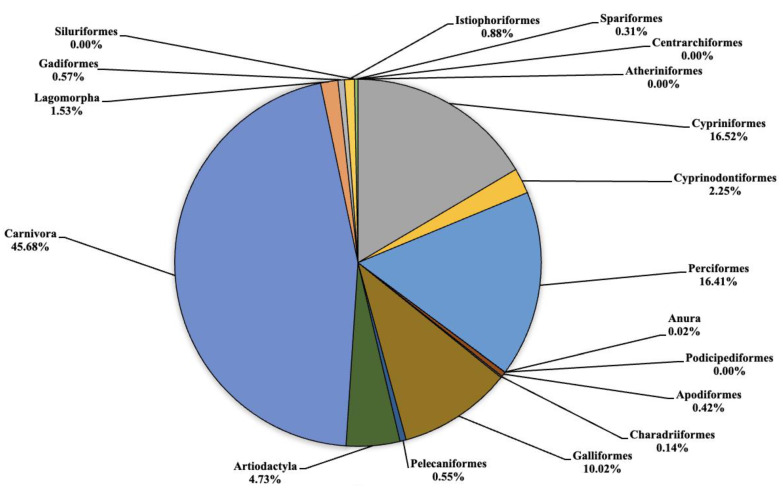
Pie chart (expressed as a percentage) representing the taxonomic orders identified in Lake Piana.

**Figure 4:**
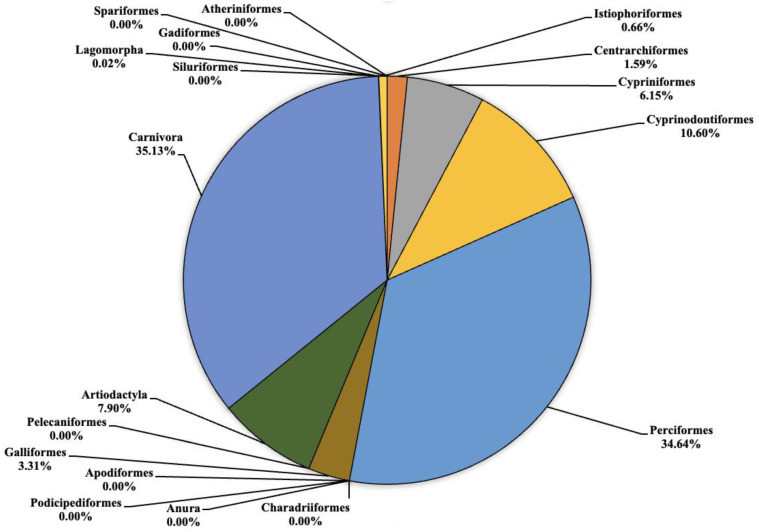
Pie chart (expressed as a percentage) representing the taxonomic orders identified in Lake Scanzano.

The analysis carried out on the eDNA samples obtained from the three Sicilian lakes showed an average number of fragment readings equal to: 124,781, 99,534 and 106,403 from Lake Poma, Lake Piana and Lake Scanzano, respectively. The data obtained were subsequently processed and cleaned to extrapolate only the fragments of interest to the study. Analysis allowed for taxonomic discrimination from the phylum to species level, with the most comprehensive results achieved at the order level. Table 2 shows the data relating to the number of fragments identified for each order in each lake analyzed. Total cleaned frequencies used to analyze the taxonomic order were 7247, 10,010.7 and 8052.5 for Lake Poma, Lake Piana and Lake Scanzano. These data are detailed and described as percentages for each site in the pie charts shown in Figure 2, Figure 3 and Figure 4, highlighting differences or similarities found between the sites.

Regarding Lake Poma (Figure 2), the highest number of fragments concerned the orders of Atheriniformes (3.61%), Centrarchiformes (9.88%), Perciformes (7.31%), Galliformes (7.46%), Artiodactyla (2.12%) and Carnivora (66.43%). For the orders of Cypriniformes, Anura, Lagomorpha, Gadiforme and Istiophoriformes, no eDNA fragments were identified. Very low quantities of fragments were detected for Cyprinodontiformes, Apodiformes, Charadriiformes and Spariformes, recorded as 0.5 (0.01%), 10.5 (0.03%), 2 and 28 (0.3%), respectively. Finally, for the orders of Podicipediformes, Pelecaniformes and Siluriformes, low but noteworthy levels were found in the number of fragments, which were 37.5 (0.52%), 65.5 (0.90%) and 88 (1.21%), respectively.

In Lake Piana (Figure 3) the highest number of fragments concerned the orders of Cypriniformes, Cyprinodontiformes, Perciformes, Galliformes, Artiodactyla and Carnivora that were equal to 1653.3 (16.52%), 225 (2.25%), 1642.3 (16.41%), 1003 (10.02%), 473.3 (4.73%) and 4572.7 (45.68%), respectively. No eDNA fragments were identified for the orders of Atheriniformes, Centrarchiformes, Podicipediformes and Siluriformes. Very low quantities of fragments were detected for Anura, Charadriiformes and Spariformes at 1.7 (0.02%), 14 (0.14%) and 30.7 (0.31%), respectively. Finally, for the orders of Apodiformes, Pelecaniformes, Gadiformes and Istiophoriformes, low but noteworthy levels were found regarding the number of fragments at 42.3 (0.42%), 54.7 (0.55%), 56.7 (0.57%) and 88.3 (0.88%), respectively. On the other hand, some orders detected in Lake Piana, such as Cyprinifromes, Anura, Lagomorpha, Gadiformes and Istiophoriformes, were not detected in Lake Poma.

As regards to Lake Scanzano (Figure 4), the highest number of fragments concerned the orders of Cypriniformes, Cyprinodontiformes, Perciformes, Galliformes, Artiodactyla and Carnivora, which were equal to 495 (6.15%), 853.5 (10.60%), 2789 (34.64%), 266.5 (3.31%), 636 5 (7.90%) and 2829 (35.13%), respectively. Furthermore, in most cases, the number of fragments obtained was even greater than those obtained in Lake Piana. No eDNA fragments were identified for the orders of Atheriniformes, Anura, Podicipediformes, Apodiformes, Charadriiformes, Pelecaniformes, Gadiformes, Siluriformes or Spariformes. Lower amounts of fragments were found for Centrarchiformes and Istiophoriformes at 128 (1.59%) and 53 (0.66%) respectively. In conclusion, for Lagomorpha, the lowest quantities of fragments were detected, which was equal to two (0.02%). Compared to the other two lakes, there were a greater number of orders for which no eDNA fragments were identified. Several absent orders were found to be present in the other two lakes, such as Pelecaniformes, Artiodactyla, Apodiformes and Charadriiformes. An absence of Atherinifromes, Podicipedifromes and Siluriformes orders was also found in Lake Piana. The same was observed for Anura and Gadiformes when compared to Lake Poma.

**Figure 5:**
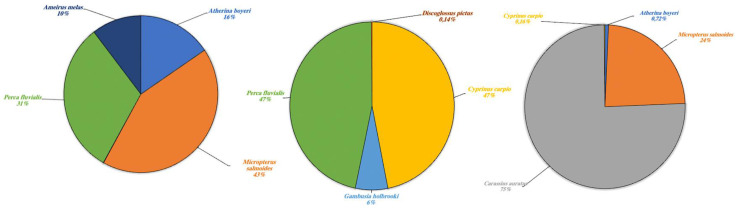
Aquatic species detected in Lake Poma (**left**), Lake Piana (**center**) and Lake Scanzano (**right**) using eDNA analysis. The results are expressed in percentages of DNA fragments obtained.

**Figure 6:**
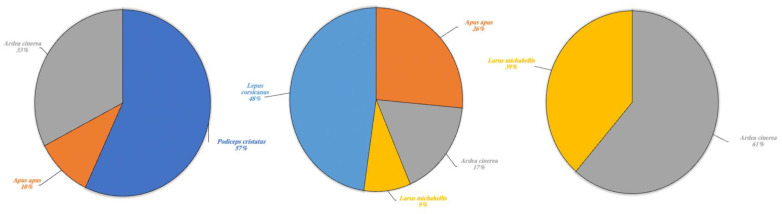
Other wild species detected in Lake Poma (**left**), Lake Piana (**center**) and Lake Scanzano (**right**) using eDNA analysis. The results are expressed in percentages of DNA fragments obtained.

**Figure 7:**
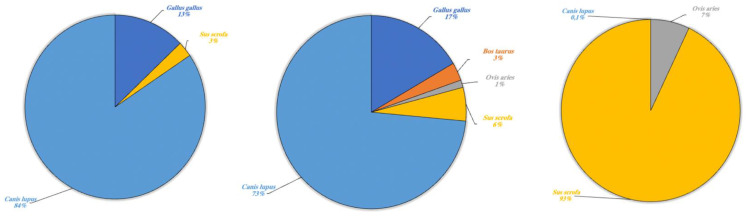
Domestic terrestrial species detected in Lake Poma (**left**), Lake Piana (**center**) and Lake Scanzano (**right**) using eDNA analysis. The results are expressed in percentages of DNA fragments obtained.

**Figure 8:**
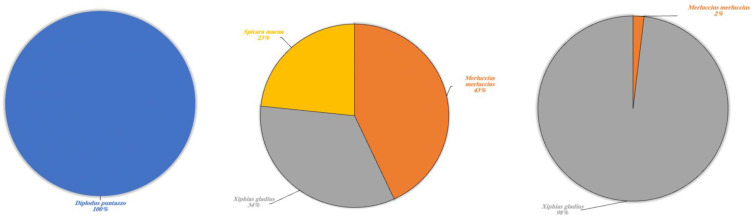
Marine species detected in Lake Poma (**left**), Lake Piana (**center**) and Lake Scanzano (**right**) using eDNA analysis. The results are expressed in percentages of DNA fragments obtained.

In various cases, environmental DNA analysis led to the identification of the species present within different orders. Our results highlighted the possibility to identify different types of species indicated in Table 3 and divided these into four categories: wild aquatic species (*sensu strictu*), other wild species, domestic terrestrial species and marine species. The latter has been included in a separate category as the lakes collect freshwater and are not in communication with any marine environment, thus the presence of marine species was an expected result. Each single category is described respectively (Figure 5, Figure 6, Figure 7 and Figure 8).

Results for aquatic species are detailed in Figure 5 and expressed as a percentage of fragments obtained. The highest fragment percentages in Lake Poma were obtained for species *Micropterus salmoides*, *Perca fluviatilis*, *Atherina boyeri* and *Ameirus melas* (43%, 31%, 16% and 10%, respectively). High fragment percentages in Lake Piana were observed in *P. fluviatilis* (47%*)* only. *Discoglossus pictus*, *Cyprinus carpio* and *Gambusia holbrooki*, not found in Lake Poma, showed fragment percentages of 0.14%, 47% and 6%, respectively. Finally, regarding Lake Scanzano, the highest fragment percentage was identified for *Carassius auratus* (75%), a species seemingly not present in the other two lakes. However, similar to Lake Poma, fragments of *M. salmoides* and *A. boyeri* were identified, albeit at lower percentages (24% and 0.72%, respectively), and similar to Lake Piana, fragments of *C. carpio* were found, although, once again, at much a lower percentage (0.16%).

Other wildlife species for which eDNA fragments were detected in the three lakes are shown in percentages in Figure 6. A high fragment percentage were detected in Lake Poma for *Podiceps cristatus* (57%) and limited fragment percentages for *Apus apus* and *Ardea cinerea* at 10% and 33%, respectively. In Lake Piana, the highest fragment percentages concerned the species *Lepus corsicanus* (48%) and *A. apus*, although this last species was found at a higher rate (26%) than in Lake Poma. Similar to Lake Scanzano, fragments of *A. cinerea* and *L. michahellis* were also detected. In Lake Piana, however, detection rates were lower (17% and 9%, respectively), whilst in Lake Scanzano, these two species were the only fragments identified (61% and 39%, respectively).

Regarding domestic terrestrial species (Figure 7) in Lake Poma, *Canis lupus* (84%) was detected with the highest fragment percentage and *G. gallus* (13%) and *S. scrofa* (3%) with the two lower rates.

Likewise, in Lake Piana, the highest fragment percentage was recorded for *C. lupus* (73%) and lower percentages for *G. gallus* (17%) and *S. scrofa* (3%). However, fragments of *B. taurus* and *O. aries* were also detected in Lake Piana at 3% and 1%, respectively. Lastly, in Lake Scanzano, a high fragment percentage was detected only for *S. scrofa* (93%), with lower percentages recorded for *O. aries* and *C. lupus* at 7% and 0.1%, respectively.

To conclude, a fascinating result concerned the detection of eDNA fragments of typically marine species (Figure 8). In Lake Poma, *Diplodus puntazzo* was detected at a rate of 100%, whilst *Merluccius merluccius* and *Xiphias gladius* were detected in Lake Piana and Lake Scanzano at differing percentages. In more detail, rates were found to be 43% and 34%, respectively, in Lake Piana and 2% and 98% in Lake Scanzano. Moreover, fragments of *Spicara maena* (23%) were only detected in Lake Piana.

**Figure 9:**
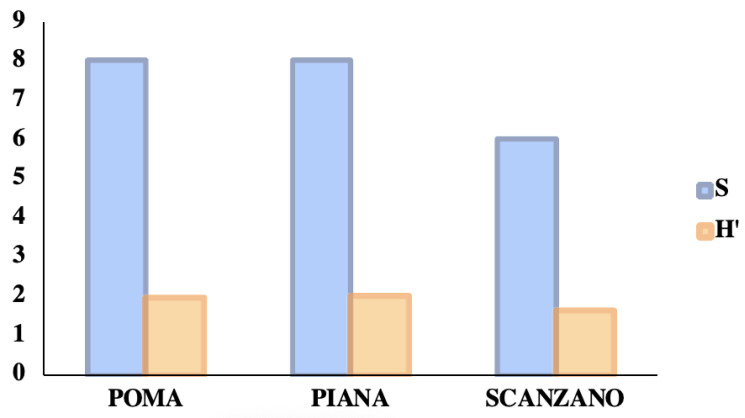
A comparison between the number of taxa and the values of the biodiversity index (relative to wild vertebrates) in three lakes. In detail, the specific richness values (S) and the biodiversity indices (H′) calculated with the Shannon algorithm.

Figure 9 shows the number of taxa and the values of the biodiversity index (relative to wild vertebrates) found in the three lakes examined and compared. Lake Piana was the lake with the highest specific richness and biodiversity values, while Lake Scanzano was found to be the lowest.

Despite the similarity of these values, the coenoses of the three lakes were found to be quite different from each other. In fact, by comparing the similarity values found, there are differences of at least 50% from a qualitative point of view and at least 60% from a qualitative–quantitative point of view (Table 4).

Regarding the environmental typologies around the three lakes, Table 5 shows the number of habitats found and the relative diversity values.

Once again, the values were quite similar to each other. However, unlike the data observed regarding the fauna, habitat comparison yielded a value of less than 50% similarity only in the case of the Bray–Curtis index (Table 6).

## 4. Discussion

Biomonitoring is essential to analyze the biological diversity, contamination and ecological status of the ecosystems examined [53,54,55]. Among the different approaches, one of the most important in recent years is based on the detection and characterization of DNA released by organisms into the environment and that are classified into two types: organismic and extraorganismic [56,57]. 

In this study, the environmental DNA technique was applied to three Sicilian lakes in order to provide the first snapshot of vertebrate biodiversity in these three lakes that would be useful to create a baseline of knowledge regarding the biodiversity in these three lakes. Our preliminary eDNA results showed differences between the three lakes, in contrast with high similarity found in the composition of surrounding habitats, an issue that will need to be further investigated. Regarding aquatic orders, fragments of Cypriniformes, Cyprinodontiformes, Perciformes, Centrarchiformes and Atheriniformes were identified, although detected in differing abundances in the three sites. Similar results were observed by analyzing the number of fragments of other orders. In regards to the orders and wild aquatic species, we had sporadic information based on our knowledge, sightings or information collected from the local population and amateur fishermen, which reported both about the species that we identified with eDNA and also about the species that were not identified with eDNA in our study [58,59,60]. Regarding the last ones, the not revealing of their eDNA could be due to the lack of a DNA barcode in the reference libraries [61,62,63]. Despite this, these results constitute the first snapshot of the three lakes to be further explored and expanded in future sampling, also during other seasons. A particularly important result of the eDNA approach concerned the ability to verify the presence of species that were not strictly aquatic, however dependent on these ecosystems to some extent. Species such as *Podiceps cristatus* and *Ardea cinerea*, which nest in these environments, *Apus apus*, a species which uses the reservoirs for drinking, or *Larus michahellis,* which is present all year round, were identified. However, this is a relatively low number of species compared to those actually present. This fact depends partially on the sampling period but also on the phenology of the species. Bird species (both migratory and sedentary) in these areas are, in reality, much more numerous, not to mention the known presence of amphibians and reptiles not yet detected by eDNA. Indeed, during sampling, bird species such as *Egretta garzetta*, *Bubulcus ibis*, *Anas plathyrhyncos*, *Gallinula chloropus*, *Actitis hypoleucos* and *Ardea alba* were observed, and reptiles, such as *Natrix helvetica* [64], or amphibians, such as *Xenopus laevis* or *Bufo bufo* [65,66,67], as well as being known to the area, were also seen during sample collection. 

The different biodiversity values could be due to a number of factors, such as water sampling (e.g., the need to increase sampling to cover a wider surface area) or the degradation processes of the DNA released into the environment. Indeed, it is important to consider that environmental DNA is a heterogeneous mixture of genetic materials, including chromosomes and plasmids protected inside cells, or other types of cellular debris and extracellular DNA fragments that are floating in the environment [68]. It has also been observed that the methods of preservation and extraction of the sample can also influence the final result [34,69,70,71,72]. Regardless of the nature of the DNA released into the environment, it is clear that its fate can differ as it encounters factors and/or conditions which either protect it and keep it intact or degrade it [73,74,75,76,77]. It is known rather that the resistance of environmental DNA in water samples depends on the characteristics of the molecule (length, conformation, sequence) and on environmental characteristics [78]. For example, environmental temperatures or salinity conditions, or the availability of oxygen, ultraviolet or solar radiation, can also influence the degradation of the molecule through denaturation processes [79,80,81,82,83,84,85,86,87,88,89]. Even microbial communities and extracellular enzymes can influence the degradation processes [90].

Abiotic and biotic conditions of the studied ecosystem could also have an influence on the performance of the primer, which can vary under differing conditions [91,92]. In addition, studies on target 16s sequencing of mock communities reported large deviations from expected values, dependent on sequencing primers, extraction methods and the sequencing platform applied [93]. The choice of primer pairs that allow DNA amplification of specific taxonomic groups and discriminate between species is crucial. A large variety of primer pairs have been developed, either universally or specifically, to amplify target clades [94,95,96,97]. Multiprimer comparison generally found considerable differences in the amplified taxonomic specificity and species discrimination power both in silico and in situ [98]. Thus, to increase the ability of species detection, it would be appropriate to use different primer pairs in combination [99].

To implement DNA metabarcoding for the identification of species and ecosystem biomonitoring, we need reliable sequence reference libraries of the known taxa [61]. Currently, the incompleteness of DNA barcode reference libraries represents a significant limit to unveiling total biodiversity, especially of an aquatic ecosystem and species from lakes. Among aquatic taxa, species-rich groups, such as arthropods and polychaetes, or economically important fish are better represented in libraries, while specific taxonomic groups, at the local/regional level in particular, are completely absent [61,62,63]. Results of metagenomic analyses highlighted the presence of different categories of species. Some of these concerned the category of wild aquatic species in the strict sense, typical of these sites. The ability to use eDNA to detect species of fish present in freshwater environments certainly offers new possibilities for less invasive censuses and for the creation and/or updating of regional fish maps. However, an extremely important result regarded the acquisition of information on other wild species or domestic terrestrial species. As far as the presence of domestic species is concerned, this could be due, in some cases, such as *Bos taurus*, *Ovis aries* and *Canis lupus familiaris*, to the shepherds’ habit of keeping these species near to reservoirs for water supply. In other cases, the presence of eDNA, such as that of *Gallus gallus* and *Sus scrofa*, linked to anthropic activities or farming in the surrounding area, could reach the water body by soil leaching or through discharges into small watercourses that flow into the reservoirs [100,101,102].

On the other hand, the presence of marine fish species, typically used for human nutrition, could only be explained by waste disposal in these waters. This could be due to anthropic impacts that influence freshwater environments today [103,104], although fisheries sector businesses would seem to be absent from the area. Thus, differences between the three lakes, to our knowledge, are not correlated to the presence of particular anthropic activities nearby. Taken together, our results highlighted the ability to use environmental DNA evaluation not only as a tool for biodiversity census but also as a tool to evaluate the ecological status of aquatic environments. Our results confirmed that this technique has the potential to complement traditional methods, although using both approaches may offer a more comprehensive understanding of the ecosystem [105].

## 5. Conclusions

The results of this study confirmed the considerable potential of environmental DNA analysis as a tool to evaluate not only the species in a given site but also its ecological status. Results presented in this study show that DNA release into the environment could be useful to identify both strictly aquatic species and terrestrial species that use these sites as a source of water supply. Moreover, our results showed that the use of eDNA can be inserted in a much broader context than a simple census, i.e., in the evaluation of the ecological status of the ecosystem in question. It seems, for example, that environmental DNA could allow us to identify the presence of anthropogenic impacts from the illegal dumping of fishing waste. In this regard, our results detected marine species which could not be in these lakes otherwise, as the water bodies are not in communication with the marine environment. Despite this, our results confirmed that the exclusive use of this technique to replace conventional techniques entirely is not yet possible. There are still too many variables that influence the persistence of environmental DNA, and the detection or otherwise of some species cannot necessarily be connected to their absence or presence. Another important issue concerns the standardization of protocols used due to considerable deviations from the expected values, which are largely dependent on extraction methods, specificity sequencing primers and the sequencing platform applied.

Moreover, it is worth noting that our results could depend on a series of factors, including the lack of DNA barcoding of different taxa in the reference libraries, on the selection of appropriate primer pairs or on the need to use more than one pair to detect a wide range of taxa.

Conventional taxonomic skills today cannot be replaced entirely if we are to ensure the correct characterization of the biodiversity of a given site. However, results obtained undoubtedly provide a satisfactory starting point for the creation of a fish map of these sites and for a census of the biodiversity present, that can be broadened by carrying out further samplings in different seasons.

## Figures and Tables

**Table 1:** Different types of land use (Corine Biotopes; carta HABITAT 1:10.000″ of the Regione Siciliana) and their surface in hectares by applying a buffer of 1 km with respect to the perimeter in three Sicilian lakes.

	Piana	Poma	Scanzano
22.1 Small artificial reservoirs without or poor in vegetation (Phragmitio-Magnocaricetea)	0.5	3.1	2.1
31.81 Forest edge shrub communities (Rhamno-Prunetea, *Prunetalia spinosae*)	15.6	0.0	1.0
31.8A Sub-Mediterranean thermophilic shrublands with *Rubus ulmifolius*	0.0	0.9	0.0
32.211 Low maquis with *Pistacia lentiscus* and/or *Olea europaea* var. sylvestris	0.0	1.7	0.0
32.215 Shrub communities with *Calicotome villosa* and/or *C. infesta*	0.0	2.1	0.0
32.22 Euphorbia dendroides scrub (Oleo-Euphorbietum dendroidis s.l.)	11.4	0.0	0.0
32.A *Spartium junceum* shrublands	3.9	0.0	2.9
34.36 Mediterranean and sub-Mediterranean thermo-xerophilous pastures	63.2	0.0	3.3
34.5 Annual Mediterranean dry meadows (Thero-Brachypodietea)	0.0	157.3	0.0
34.6 Grasslands with perennial species (Lygeo-Stipetea)	0.0	24.5	19.2
34.633 *Ampelodesmos mauritanicus* grasslands (Lygeo-Stipetea, Avenulo-Ampelodesmion mauritanici)	91.7	13.1	29.4
34.74 Dry meadows of the central and southern Apennines	14.3	0.0	0.0
34.81 Sub-nitrophilous dry meadows with post-cultured vegetation (*Brometalia rubenti*-*tectori*)	62.2	50.3	29.6
38.11 Mesophilous grasslands with *Cynosurus cristatus* and *Lolium perenne* (*Cirsetalia vallis*- *demonis*)	56.0	0.0	0.4
41.732 Deciduous oak forests of the *Quercus pubescens* cycle (*Quercetalia ilicis*)	25.1	9.6	188.4
44.1273 Salix pedicellata woodlands (*Populetalia albae*)	16.7	9.0	8.1
44.614 Populus alba woodland (*Populetalia albae*)	1.5	0.4	5.9
45.215 Quercus suber woods (Erico-*Quercion ilicis*)	0.0	0.0	11.3
45.31A Woods with *Quercus ilex* (Quercetalia ilicis)	3.9	0.0	0.0
53.11 Phragmites australis hygro-hydrophilic communities (Phragmition)	37.4	0.0	28.2
53.62 Arundo donax hygrophilous community (Arundini-Convolvuletum sepium)	0.0	9.7	0.0
61.3B Glareicolous communities of thermophilic breccias (*Euphorbion rigidae*)	6.6	0.0	0.0
62.14 Calcareous cliff vascular communities (Dianthion rupicolae, *Polypodion serrati*)	8.1	0.0	0.5
82.12 Open field horticulture	0.0	1.0	0.0
82.3 Arable land and extensive herbaceous crops	597.0	400.7	339.2
82.3A Complex agricultural systems	151.9	19.1	14.1
83.112 Intensive olive groves	17.6	48.0	5.5
83.15 Orchards	14.9	39.6	14.7
83.16 Citrus groves	0.0	37.4	0.0
83.211 Associated vineyards (with olive groves, etc.)	0.0	0.0	0.6
83.212 Intensive vineyards	14.8	377.3	89.2
83.31 Reforestation mainly of conifers (genera Pinus, Cupressus, Cedrus, etc.)	82.9	15.5	10.8
83,322 Reforestation with a prevalence of *Eucalyptus* sp. pl.	35.8	29.1	72.4
83.325 Other reforestation or hardwood plantations	0.0	0.0	29.7
85.5 Recreational and sports areas	0.0	2.3	0.0
85.6 Cemeteries	1.6	0.0	0.0
86.12 Sparse residential fabric	2.3	0.0	1.2
86.22 Rural buildings	5.7	1.0	2.5
86.31 Industrial and/or craft and/or commercial settlements and associated spaces	17.9	2.6	9.1
86.32 Establishment of large service facilities	2.3	4.8	5.2
86.41 Quarries	5.5	4.9	0.0
86.42 Vegetation of ruderal areas and landfills	0.0	4.9	0.0
86.43 Main road arteries	2.4	0.0	8.6

**Table 2:** Taxonomic order identified by analyzing eDNA. Average final-fragments read for each order were reported for each lake. The total number of cleaned frequencies per lake was also reported.

	Poma	Piana	Scanzano
Actinopteri; Atheriniformes	261.5	-	-
Actinopteri; Centrarchiformes	716	-	128
Actinopteri; Cypriniformes	-	1653.3	495
Actinopteri; Cyprinodontiformes	0.5	225	853.5
Actinopteri; Perciformes	529.5	1642.3	2789
Amphibia; Anura	-	1.7	-
Aves; Podicipediformes	37.5	-	-
Aves; Apodiformes	10.5	42.3	-
Aves; Charadriiformes	2	14	-
Aves; Galliformes	540.5	1003	266.5
Aves; Pelecaniformes	65.5	54.7	-
Mammalia; Artiodactyla	153.5	473.3	636.5
Mammalia; Carnivora	4814	4572.7	2829
Mammalia; Lagomorpha	-	152.7	2
Actinopteri; Gadiformes	-	56.7	-
Actinopteri; Istiophoriformes	-	88.3	53
Actinopteri; Siluriformes	88	-	-
Actinopteri; Spariformes	28	30.7	-
**Total cleaned frequencies**	7247	10,010.7	8052.5

(-) for not detected.

**Table 3:** Species identified by analyzing eDNA. Average final-fragments read for each species and for each lake were recorded. The per-lake and per-category total number of cleaned frequencies was also recorded.

	Poma	Piana	Scanzano
**Wild aquatic species (*sensu strictu*)**			
* Atherina boyeri *	261.5	-	9
* Micropterus salmoides *	716	-	295
* Carassius auratus *	-	-	942
* Cyprinus carpio *	-	1653.3	2
* Gambusia holbrooki *	1	225	-
* Perca fluvialis *	529.5	1642	-
* Ameirus melas *	176	-	-
* Discoglossus pictus *	-	5	-
**Total cleaned frequencies**	1684	3520.3	1248
**Other wild species**			
* Podiceps cristatus *	114.5	-	-
* Apus apus *	21	127	-
* Ardea cinerea *	65.5	82	846
* Larus michahellis *	-	42	533
* Lepus corsicanus *	-	229	-
**Total cleaned frequencies**	201	480	1379
**Domestic terrestrial species**			
* Gallus gallus *	731	1031	-
* Bos taurus *	-	189	-
* Ovis aries *	-	77.5	427
* Sus scrofa *	153.5	349	5658
* Canis lupus *	4814	4572.7	4
**Total cleaned frequencies**	5698.5	6219.2	6089
**Marine species**			
* Diplodus puntazzo *	84	-	-
* Merluccius merluccius *	-	170	106
* Xiphias gladius *	-	132.5	5575
* Spicara maena *	-	92	-
**Total cleaned frequencies**	84	394.5	5681

(-) for not detected.

**Table 4:** Matrix of qualitative and qualitative–quantitative similarity of the fauna from the three lakes (0 = no similarity; 1 = maximum similarity).

Sorensen	Poma	Piana	Scanzano
*POMA*	1.00		
*PIANA*	0.50	1.00	
*SCANZANO*	0.43	0.43	1.00
** Bray–Curtis **			
*POMA*	1.00		
*PIANA*	0.37	1.00	
*SCANZANO*	0.37	0.27	1.00
** Morisita **			
*POMA*	1.00		
*PIANA*	0.40	1.00	
*SCANZANO*	0.43	0.31	1.00

**Table 5:** Number of habitats (S) and the diversity values (H’) in the three lakes.

	Piana	Poma	Scanzano
** S **	30	27	28
** Shannon_H **	2.234	2.034	2.192

**Table 6:** Matrix of qualitative and qualitative–quantitative similarity of the habitat of the three lakes (0 = no similarity; 1 = maximum similarity).

Sorensen	Piana	Poma	Scanzano
*PIANA*	1.00		
*POMA*	0.60	1.00	
*SCANZANO*	0.83	0.62	1.00
** Bray–Curtis **			
*PIANA*	1.00		
*POMA*	0.46	1.00	
*SCANZANO*	0.51	0.54	1.00
** Morisita **			
*PIANA*	1.00		
*POMA*	0.69	1.00	
*SCANZANO*	0.84	0.74	1.00

## Data Availability

Non-public data for privacy, contact the authors.
